# Remote photoplethysmography with constrained ICA using periodicity and chrominance constraints

**DOI:** 10.1186/s12938-018-0450-3

**Published:** 2018-02-09

**Authors:** Richard Macwan, Yannick Benezeth, Alamin Mansouri

**Affiliations:** Le2i UMR6306, CNRS, Arts et Métiers, Univ. Bourgogne Franche-Comté, 21000 Dijon, France

**Keywords:** Remote photoplethysmography, Constrained independent component analysis, Semiblind source separation, Periodicity constraint, Chrominance constraint

## Abstract

**Background:**

Remote photoplethysmography (rPPG) has been in the forefront recently for measuring cardiac pulse rates from live or recorded videos. It finds advantages in scenarios requiring remote monitoring, such as medicine and fitness, where contact based monitoring is limiting and cumbersome. The blood volume pulse, defined as the pulsative flow of arterial blood, gives rise to periodic changes in the skin color which are then quantified to estimate a temporal signal. This temporal signal can be analysed using various methods to extract the representative cardiac signal.

**Methods:**

We present a novel method for measuring rPPG signals using constrained independent component analysis (cICA). We incorporate a priori information into the cICA algorithm to aid in the extraction of the most prominent rPPG signal. This a priori information is implemented using two constraints: first, based on periodicity using autocorrelation, and second, a chrominance-based constraint exploiting the physical characteristics of the skin.

**Results and conclusion:**

Our method showed improved performances over traditional blind source separation methods like ICA and chrominance based methods with mean absolute errors of 0.62 beats per minute (BPM) and 3.14 BPM for the two datasets in our inhouse video database UBFC-RPPG, and 4.69 BPM for the public MMSE-HR dataset. Its performance was also better in comparison to other state of the art methods in terms of accuracy and robustness. Our UBFC-RPPG database is also made publicly available and is specifically aimed towards testing rPPG measurements.

## Background

Photoplethysmography (PPG) is a technique of measuring the variation in the absorption of light by human skin, first introduced by Hertzman in 1937 [1]. The experiment comprised of placing a finger between a photoelectric cell and a light source. Then on, PPG has been ubiquitously used for heart rate measurements since it is easy to use, low-cost and non-invasive. Even more non-invasive is the technique of remote photoplethysmography, henceforth referred to as rPPG, which aims at measuring the same parameters as PPG remotely, i.e. without any contact.

Verkrussysse et al. [2] showed that remote PPG signal extraction could be performed by using a simple consumer level camera. Their work postulated that the G channel of the RGB temporal traces contained the most prominent photoplethysmographic signal. These RGB temporal traces were obtained by quantifying frame-wise skin pixel data, for instance by spatial averaging, and then concatenating them. The feasibility of rPPG in a medical scenario, for patients in the neonatal intensive care unit, has also been investigated in [3]. They corroborated the ability of rPPG to obtain a signal strong enough to extract the heart rate of all the infant subjects, measured from manually selected regions of interest (ROIs). One of the paradigms in ongoing research is centered around using simple web cameras to extract clean rPPG signals employing blind source separation (BSS) techniques. Independent Component Analysis (ICA), a very common BSS algorithm has been used in several works [5–7].

ICA is a technique used to decompose a multivariate signal into the constituent signals under the assumption that the input signals be uncorrelated [8]. In this context, rPPG signal extraction can be formulated as a signal separation problem where the periodic cardiac pulse, manifested as minute chromatic variations of the skin color, is linearly mixed into the temporal traces obtained from the video data from cameras.

Let $\mathbf{s}=(s_{1},s_{2},...,s_{n})^{T}$ be the time varying color traces from *n* channels, obtained by linear mixing of *m* independent source signals denoted as $\mathbf{c}=(c_{1},c_{2},...,c_{m})^{T}.$ The linear mixing process is then expressed as $\mathbf{s}=\mathbf{Ac},$ where the linear memoryless mixing of the channels is represented by the matrix $\mathbf{A}_{n\times m}.$ ICA aims to obtain the linear unmixing matrix $\mathbf{W}_{m\times n}$ to recover all the independent components with minimum knowledge of **A** and **c**. The separated components ${\mathbf{y}}=(y_{1},y_{2},...,y_{m})^{T},$ are obtained by $\mathbf {y}=\mathbf {Wx}$ [9].

This linear formulation of ICA suffers from two inescapable ambiguities [10, 11]. First, the independent components cannot be obtained in a deterministic order. The same independent components can be obtained by a different permutation of the columns of $\mathbf {W}.$ Second, the independent components cannot be obtained to the exact amplitude and sign.

In addition, with respect to the task of rPPG signal measurement, the nature of the required component is not entirely unknown. That is to say, we are not entirely *blind* in this case. Excluding scenarios that have periodic movements, like fitness-based applications, it is evident that the most periodic signal, linearly mixed into the RGB temporal traces, must correspond to the cardiac pulse. Furthermore, we only require one component, viz., the rPPG pulse signal, which simplifies the optimization process and changes the weighting matrix from $\mathbf{W}_{m\times n}$ to the vector $\mathbf{w}_{m\times 1}.$ This condition of extracting a single component is not uncommon and is perceived in various biomedical scenarios. The On–Off simulation scheme of an fMRI experiments [9] is one such example. As a consequence, incorporation of such a priori knowledge into the component extraction algorithm can help in alleviating the indeterminacy issue.

**Fig. 1:**
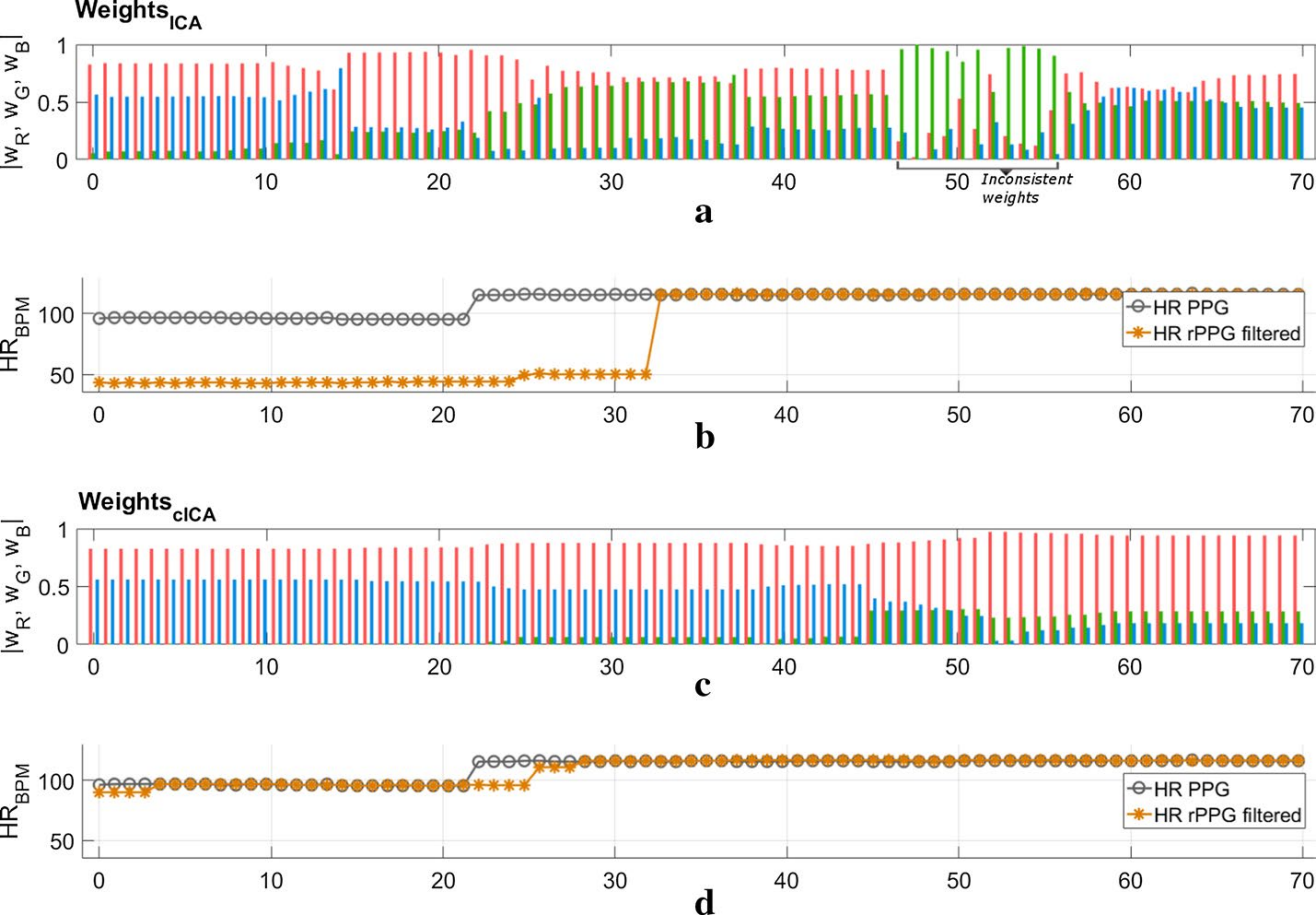
Window-wise weight analysis showing ICA weights and HR in **a** and **b** vs cICA weights and HR in **c**, **d**. For the exact same data, different values of $\mathbf {w}=|w_R,w_G,w_B|$ corresponding to the RGB channels can extract an accurate rPPG signal. Absolute values of the weights are shown for concise display

To inspect this incorporation of a priori information, we performed analysis of the ICA weighting vectors that extract the best component. We used a challenging video for this analysis where ICA fails to extract the best rPPG signal continuously as is visible in the first 30 s of Fig. 1b. The weights extracted using the cICA algorithm proposed later in this paper are also shown in Fig. 1c for comparison. Acknowledging the ability of the cICA algorithm to extract the more difficult rPPG signal better than ICA, we would like to emphasize on the later part of the video from the 30 s mark where ICA is able to extract the rPPG signal successfully. It is evident from the figure that the ICA weights, although extracting the rPPG signal correctly, are prone to abrupt changes, as seen around the 50 s mark.

Furthermore, the fact that rPPG extraction is successful even with these abruptly changing weights, points to the existence of several solutions. This is where the blindness of ICA is evident, i.e. a given rPPG signal can be extracted using different weights. This lack of a unique solution, added by the problem of indeterminacy in scale and permutation of the weighting matrix, makes ICA weights estimation an ill-posed problem. Thus, augmenting the ICA algorithm with *constraints* in a systematic and flexible manner to frame a constrained independent component analysis (cICA) algorithm is a promising course of action. In this paper, we present the formulation of this cICA algorithm to restrict the solution space in order to extract the rPPG signal more uniformly even for videos with challenging conditions with respect to light and motion variability.

Specifically, we propose to improve the ICA formulation by making three modifications. First, since we require only one component, the problem of component *separation* becomes that of component *extraction*. Second we are not entirely blind about the nature of the required rPPG signal, which is quasi-periodic. Adding a priori information about this quasi-periodicity, helps to restrict the solution space while maintaining our direction of search towards the weights for the best rPPG signal. The cICA algorithm avoids the ambiguities of ICA by directly converging to the best independent component, aiming to eliminate the problem of multiple optima. Finally, we incorporate a priori information about the biophysical properties of the skin, using constraints based on its tone, to steer the optimization in the direction of choosing the correct blood volume pulse. To the best of our knowledge, the incorporation of constraints based on the periodicity and physical properties of the skin within an optimization framework, and the impact of their combination on the quality of the rPPG signal, has not been attempted yet in literature.

A summary of the related work is given in “Previous work” section followed by the formulation of the periodicity measure and the chrominance constraints in “Proposed method” section. In “Results and discussion” section we validate our algorithm against our in-house database, UBFC-RPPG which comprises of two datasets of 9 and 46 videos respectively. This database will be made publicly available along with the ground truth.^1^ To the best of our knowledge, this is the first publicly available dataset focusing on the application of rPPG analysis.

## Previous work

One of the pioneer works that used ICA for rPPG measurements comprised of using RGB temporal traces from a simple web camera to extract the cardiac pulse, which although proving the basic idea, was not very robust against motion artifacts [2]. The advantage of ICA for rPPG measurements over principal components analysis (PCA), autocorrelation and cross-correlation was also investigated in [12]. McDuff et al. further investigated the usage of more color channels with a five band (RGBCO) camera and postulated that better rPPG signals were obtained using the GCO channels rather than traditional RGB channels [5]. In another work, Wang et al. performed ICA on pixel based rPPG sensors, using motion compensated pixel-to-pixel pulse extraction based on optical flow vectors, spatial pruning and temporal filtering to obtain a robust pulse signal [13].

In contrast to the blind source separation methods, De Haan et al. have exploited the effect of light on human skin due to its unique physical characteristics, introducing chrominance based methods [4]. The chrominance (CHROM) signals are generated from the RGB traces with the use of a skin-tone standardized linear combination compatible with different skin colors. The chrominance based methods assume that the light reflected from skin generally occupies similar coordinates in the RGB space under white illumination. This direction of the standard skin tone was experimentally estimated using their in-house database.

They further advanced upon their chrominance based methods proving that the various absorption spectra of arterial blood manifest along a specific vector in a normalized RGB space, termed as the blood volume pulse vector [14]. Recently, they introduced a mathematical model that incorporates the relevant optical and physiological properties of skin reflection using which they proposed a new algorithm based on the Plane-Orthogonal-to-Skin (POS) which is a plane orthogonal to the skin-tone in the temporally normalized RGB space, suitable for rPPG pulse extraction [15].

Another class of methods focuses on smart ROI selection paradigms. Feng et al. propose to improve the extracted rPPG signal by performing K-means clustering on a feature space modeled to select skin ROIs corresponding to good rPPG signals [16]. Recently, Bobbia et al. used temporal superpixels, corresponding to structurally and spatially coherent regions, to extract candidate pulse signals which were then merged averaged, weighted by superpixel-wise pulsatility measures, into an rPPG signal [17]. In a similar work, Kumar et al. proposed an automatic weighting method for different tracked regions to construct the rPPG signal based on maximum ratio diversity [18], where the weights depend on the blood perfusion and incident light intensity in the region [19].

Motion and illumination disturbances are a major issue in rPPG measurements and is an active area of research. Li et al. used face tracking and Normalized Least Mean Square adaptive filtering methods to compensate against illumination variations. They also perform non-rigid motion elimination by discarding temporal segments of the signal having high standard deviation from the signal mean [20]. Recently, Butler et al. have assessed the effect of the topology and optical variations of human skin in relation to horizontal movements of the subject and showed that, in presence of motion, the quality of the rPPG signal is determined by the properties of the area of skin chosen [21]. Tasli et al. have used facial landmark locations based on active appearance models [22] to obtain a motion compensated temporal color signal, where free head movement by the subject was allowed [23].

Recently, Wang et al. have tried to improve rPPG measurements during fitness exercises, from subjects running on a treadmill. Their proposed method called Sub-band rPPG, suppresses different distortion-frequencies using independent combinations of color channels, based on the idea that the degrees of freedom of noise reduction can be increased by decomposing the n-wavelength camera signals into multiple orthogonal frequency bands. In another work, they exploit the limited variation of human relative pulsatile amplitude to design a low cost filtering method called amplitude selective filtering. The spectral amplitude of, e.g. the R channel, is used to select the frequency components in the RGB channels inside the assumed “characteristic pulsatile amplitude range” for pulse extraction, while pruning the rest of components as noise.

Interestingly, machine learning has also been investigated to obtain rPPG measurements. Osman et al. have trained a discriminative statistical model to estimate the blood volume pulse (BVP) signal from the human face using ambient light to obtain promising results. On the other hand, Alqaraawi et al. have used the automatic multi scale-based peak detection (AMPD) algorithm coupled with a Bayesian learning approach to estimate reliable heart rate variability metrics. Many new rPPG measurement algorithms have been introduced recently. An overview of a wide range of Imaging PPG (IPPG) systems has been provided by Sun and Thakur demonstrating the research on rPPG and showing its ubiquity and widespread acceptance [24]. In a similar work, McDuff et al. provide a review on state of the art PPG imaging considering measurements other than pulse rate under realistic conditions such as presence of motion artifacts [25]. Another interesting contribution was done by Tarassenko et al. by using autoregressive models to eliminate the effect of light flicker on videos of patients undergoing haemodialysis, showing the feasibility of using rPPG measurements in a relatively uncontrolled environment [26].

Incorporation of a priori information in order to aid the optimization process is also an interesting approach in signal separation. Lu et al. have used a reference signal to coax the separation process to converge towards the desired signal resembling the reference signal [9]. They use the Lagrange multipliers method using the difference between the reference signal and the estimated signal as a constraint to be minimized. In a work inspired by theirs, Tsouri et al. proposed a method of constrained ICA using a rectangular pulse as a reference signal in the framework of rPPG [27].

In an rPPG signal extraction problem, employing a sufficiently accurate reference signal is prone to the evident complication of choosing its right frequency. This can be done in two possible ways. One alternative is to repeatedly compare the extracted rPPG signal to reference signals of different frequencies, as done by Tsouri et al. [27], which as expected is computationally taxing—around 30 times slower than traditional ICA. The other alternative is to update the frequency of the reference signal continuously, in effect making it a parameter to optimize. This increases the complexity of the problem and reduces the probability of convergence. A PPG signal is a very apt reference for rPPG extraction whose synthesis depends critically on the required frequency, even more so than the actual shape of the signal.

To avoid this limitation, we use autocorrelation as the a priori information for guiding the cICA separation algorithm which then chooses the most periodic component representing the blood volume pulse. To further aid the convergence, we apply chrominance-based constraints based on the standardized skin tone as used by De Haan et al. [4]. The use of two constraints increases the probability of convergence towards the best rPPG signal. As already mentioned, there are multiple paradigms for rPPG measurements such as methods based on physiological properties of the skin, smart ROIs, machine learning approach, and of course, source separation methods. This work contributes to the class of methods based on semi-blind source separation, with constraints based on periodicity and physical properties of the skin. Next, we present the proposed method and explain in detail the two constraints used.

## Proposed method

We start with the basic formulation of ICA where the required signal vector $\mathbf {y}$ of size *N* is extracted from the RGB temporal traces matrix $\mathbf{x}$ of size $3 \times n$ using a weighting matrix $\mathbf {w}$ of size $3 \times 1$ according to $\mathbf{y}=\mathbf{w}^T\mathbf{x}.$ As mentioned earlier, we aim to perform component extraction instead of separation, which is reflected in the change in the size of the weighting matrix from $3 \times 3$ in basic ICA to $3 \times 1$ for cICA. The cICA algorithm aims to optimize for the best weighting matrix $\mathbf {w},$ which maximizes the objective function while satisfying the imposed constraints.

Typical biomedical signals like ECG and PPG signals are known to be periodic or semi-periodic. This implicit property of periodicity of biomedical signals can be exploited to guide the component extraction process in converging to the component with the highest periodicity. Accordingly, we use autocorrelation as one of the constraints to nudge the algorithm towards selecting components having periodicity higher than a given threshold. Furthermore, we also incorporate constraints based on the physical properties of the skin, which directly effect the light reflected from the skin, and consequently the color or *chrominance * information therein. This constraint developed using the CHROM method which is undoubtedly one of the most reliable techniques in literature [4]. These two constraints are next described in detail.

### Autocorrelation as a periodicity measure

**Fig. 2:**
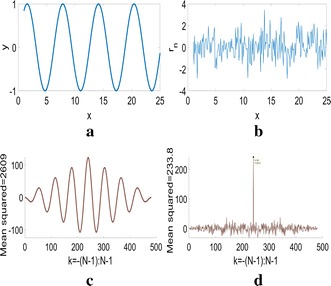
Autocorrelation of a sinusoid vs a random signal. **a** *y*_1_ = *sin(x)*, **b** *y*_2_ = *randn*(1,*N*), **c** autocorrelation of *y*_1_, **d** autocorrelation of *y*_2_

Autocorrelation is the correlation of a signal with itself at different lag times provided it is sampled at a sufficiently high frequency. For a time series signal $\mathbf {y}=[y_{1\,}y_{2} \cdots y_{N}]$ of *N* elements, its discrete autocorrelation $r_{k}$ at lags $k\in \left[-\,({\text{N}}-1) ,\ldots,\text {N}-1\right]$ is given by

1 $$\begin{aligned} r_{k}=\sum \limits _{j = 0}^{N - 1} {{y_j}}\odot \overset{{k}}{y_{j}} \end{aligned}$$

where $\overset{{k}}{y_{j}}$ is the* j*th element of the signal $\mathbf {y}$ lagged (or led for $k<0$) by *k* units and padded with zeros to the left (or right for $k<0$) and $\odot$ is the element-wise multiplication operator. A periodic signal typically has a higher correlation with itself compared to a non-periodic one. This high correlation can be quantified as the mean of the squared autocorrelation of the signal and consequently can be used as a measure of the periodicity of a signal. Figure 2 depicts the high autocorrelation of a periodic sinusoid compared to that of a uniform random signal with the mean of the squared autocorrelation is much higher than that of the random signal.

To aid the use of autocorrelation as a periodicity measure and simplify its computation, two modifications need to be made. First, since the autocorrelation is symmetric, we only compute the correlation for lags $k\in [0,\ldots,N-1].$ Second, since the correlation at lag 0 will always be high we set the autocorrelation to 0 at lag $k=0$. This is visibly prominent in Fig. 2d which exhibits a peak at lag $k=0.$ Thus, we use the autocorrelation given by $\mathbf {r}=[r_{1\,}r_{2} \cdots r_{N}]$ comprising of *N* values given by Eq. 1, with the exception of $r_{1}=0$. Keeping in mind that $r_{k}$ is a scalar, Eq. 1 can be rewritten in matrix notation as

2 $$\begin{aligned} r_{k}=\mathbf {y}[\overset{{k}}{\mathbf {y}}]^{T}=\overset{{k}}{\mathbf {y}}\mathbf {y}^{T} \end{aligned}$$

where $\overset{{k}}{\mathbf {y}}$ is again the signal $\mathbf {y}$ lagged by *k* units. Furthermore, to simplify the derivation of the autocorrelation, $\overset{{k}}{\mathbf {y}}$ can be rewritten as $\mathbf {y}T_{k}$ where $T_{k}$ is a toeplitz-like matrix that incorporates the lagging at lag *k* and padding with zeroes and is given by

3 $$\begin{aligned} T_{k}=\left[ \begin{array}{ccccccc} 0 &{} \cdots &{} 0 &{} 1 &{} 0 &{} \cdots &{} 0\\ 0 &{} \cdots &{} 0 &{} 0 &{} 1 &{} \cdots &{} 0\\ \vdots &{} \ddots &{} \vdots &{} \vdots &{} \vdots &{} \ddots &{} \vdots \\ 0 &{} \cdots &{} 0 &{} 0 &{} 0 &{} \cdots &{} 1\\ 0 &{} \cdots &{} 0 &{} 0 &{} 0 &{} \cdots &{} 0\\ \vdots &{} \ddots &{} \vdots &{} \vdots &{} \vdots &{} \ddots &{} \vdots \\ 0 &{} \ldots &{} 0 &{} 0 &{} 0 &{} \cdots &{} 0 \end{array}\right] =\left[ \begin{array}{cc} 0_{N-k,k} &{} I_{N-k}\\ 0_{k,k} &{} 0_{k,N-k} \end{array}\right] \end{aligned}$$

$T_{k}$ is an $N\times N$ matrix composed of the first $N-k$ rows made up of $(N-k)\times k$ zeroes and an identity matrix of size $N-k$. Thus, $r_{k}$ becomes

4 $$\begin{aligned} r_{k}=\mathbf {y}T_{k}\mathbf {y}^{T} \end{aligned}$$

making its differential with respect to $\mathbf {y}$ easier to calculate. One of the important requirements for the optimization framework was to utilize the first and second derivatives of autocorrelation $r_{k}$ with respect to the weighting matrix $\mathbf {w}$. This formulation, although being non-trivial, is presented in detail in the appendix to maintain continuity here.

### Chrominance based constraint

Although autocorrelation does help the optimizer to converge to a weighting matrix that extracts the correct component for simple videos, in a more realistic scenario it is prone to having not so well defined maxima. Additionally, in fitness scenarios with repetitive movements, the assumption that the most periodic component being the rPPG signal is perturbed by the periodic motion component of the fitness activity. This calls for the use of another constraint, which is not fundamentally affected by periodic components, to aid the convergence for which the CHROM constraint [4] is a suitable candidate.

To confirm this requirement, and to correlate the autocorrelation with the weights, the mean squared autocorrelation was plotted against all the possible orthonormal weights, $(w_R, w_G, w_B)$ whose components represent the contribution of each of the RGB channels in forming the rPPG signal. All these possible weight vectors in $\mathbf {R}^3$ are spanned by the standard basis $w_1 = (1, 0, 0), w_2 = (0, 1, 0), w_3 = (0, 0, 1),$ i.e.,

5 $$\begin{aligned} (w_R, w_G, w_B) = w_1 + w_2 + w_3 \end{aligned}$$

**Fig. 3:**
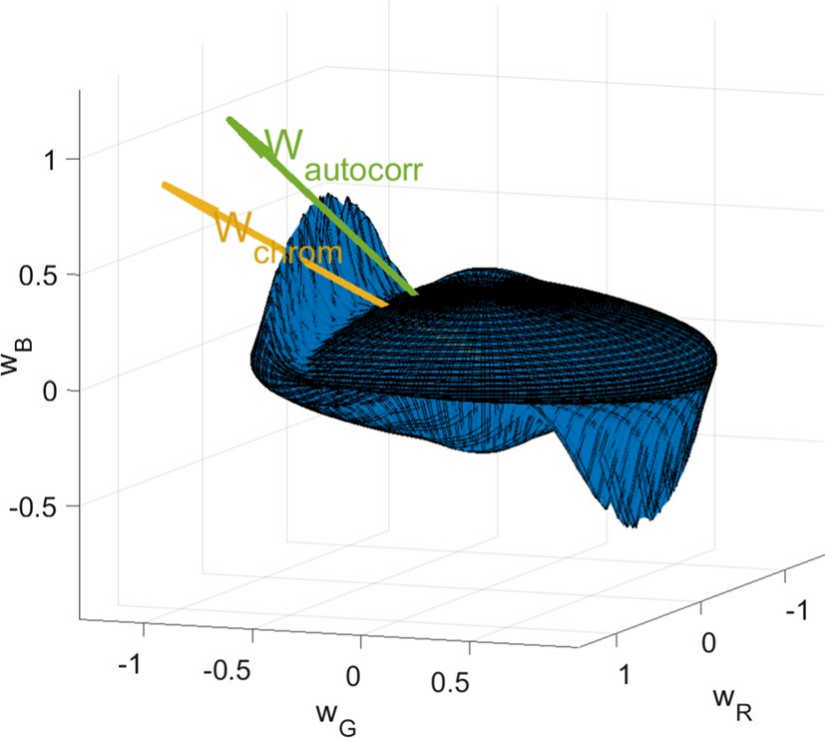
Mean-squared autocorrelation, $E\{\mathbf {r}^2\}$ vs the weighting matrix, $\mathbf {w}$

A temporal section of the RGB traces, 30 s long, $\mathbf {x}_{t}$, was used to perform this weights analysis. To plot the autocorrelation as a function of the vector space $(w_R, w_G, w_B)$, each vector *w* was scaled by the corresponding mean squared autocorrelation of $y_{t}=w\mathbf {x}_{t}$ to obtain the plot in Fig. 3. This plot can be thought of as a deformation of the unit sphere, owing to the orthonormality of the weighting vectors, by the mean squared autocorrelation of $y_t$. The ideal weighting vector giving the maximum autocorrelation is depicted as $\mathbf {w_{autocorr}}$ and the CHROM weighing vector is depicted as $\mathbf {w_{chrom}}.$

It can be seen from Fig. 3 that the mean squared autocorrelation is symmetric with respect to $\mathbf {w},$ i.e., there will always be a dual of a given weighting vector which would give the exact same autocorrelation. This is not necessarily a disadvantage since the optimizer will converge irrespective of the initial direction chosen. However, it is also visible that the plot is not very *peaky*, i.e. the maximum autocorrelation is localized to an area of smaller slope, which might make the convergence slower towards the end. It is also noteworthy that the weighting matrix corresponding to the maximum autocorrelation, $\mathbf {w_{autocorr}}$ and the CHROM weighting matrix, $\mathbf {w_{chrom}}$ depicted as vectors point around the same vicinity. In other words, the rPPG signal extracted using the CHROM method also exhibits high autocorrelation.

On the other hand, chrominance-based methods tend to be restrictive in choosing the weighting matrix based on their linear formulation. According to De Haan and Jeanne [4], a chrominance signal which incorporates the maximum photoplethysmographic information is obtained using a standardized skin tone resulting in an algorithm that can work correctly regardless the color of the illuminant. The CHROM signal is given by

6 $$\begin{aligned} S=X_f-\alpha Y_f \end{aligned}$$

where $X_f=3R_f-2G_f$ and $Y_f=1.5R_f+G_f-1.5B_f$ are the projections of the RGB traces on to standardized skin tone space and $\mathbf {x}=[R_f, G_f, B_f]^T$ is the bandpass filtered temporal RGB trace of size $3\times N.$ $\alpha$ is the ratio between the standard deviations of $X_f$ and $Y_f$ giving

7 $$\begin{aligned} S=3\left( 1-\frac{\alpha }{2}\right) R_f -2\left( 1+\frac{\alpha }{2}\right) G_f +3\frac{\alpha }{2}B_f = \mathbf {w_{chrom}x} \end{aligned}$$

where $\mathbf {w_{chrom}}$ is the CHROM weighting matrix. The goal of the proposed rPPG extraction algorithm is to converge towards the weighting matrix that simultaneously gives a component of high periodicity and is within the vicinity of the weights $\mathbf {w_{chrom}}$ up to a certain threshold. An analysis of the effect of the combination of these two constraints is presented in the next section.

### Combination of periodicity and chrominance based constraints

**Fig. 4:**
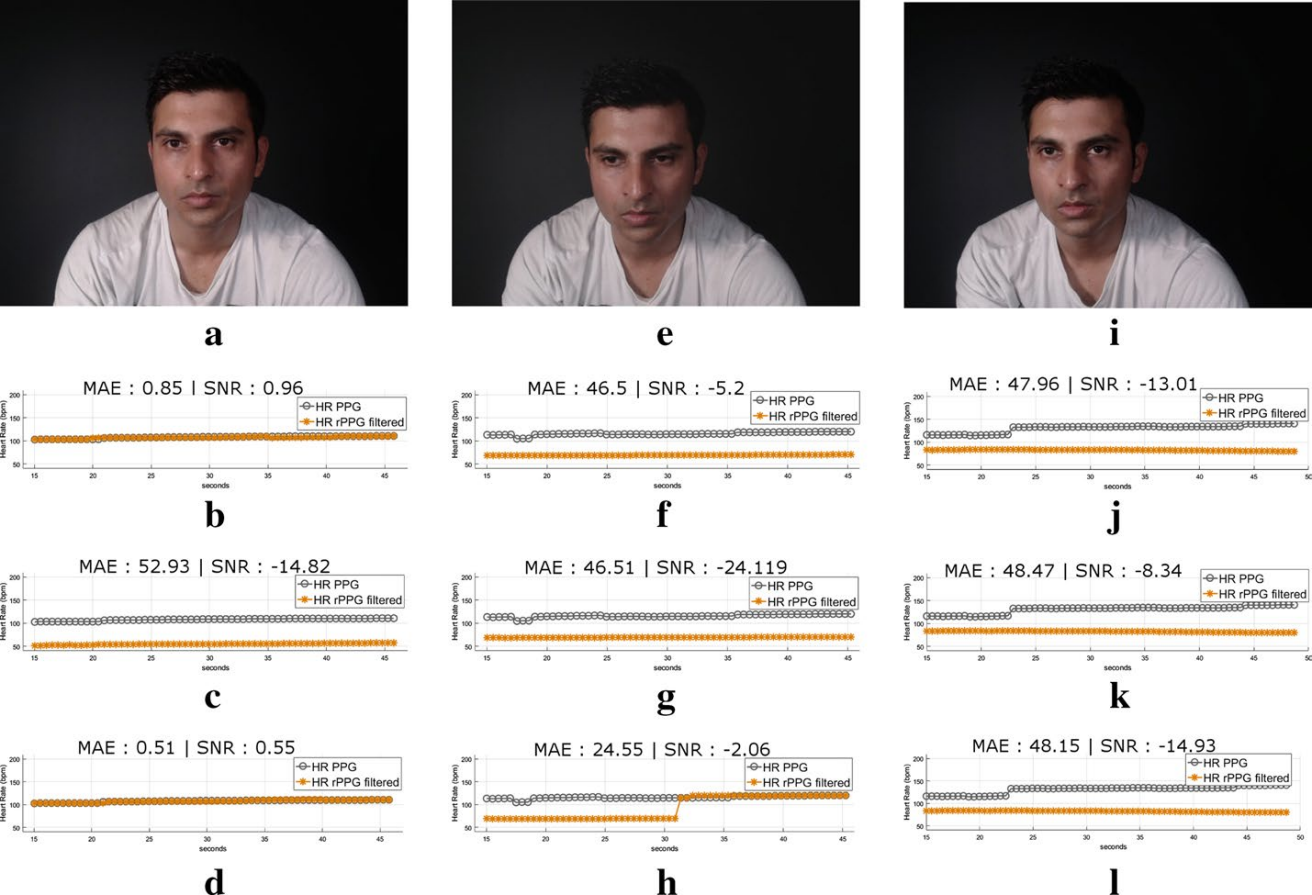
Utility of the combination of periodicity and chrominance constraints in a fitness scenario. **a** LIGHT, **b** CHROM, **c** CICA with periodicity constraint, **d** CICA with periodicity and chrominance Constraints, **e** MEDIUM, **f** CHROM, **g** CICA with periodicity constraint, **h** CICA with periodicity and chrominance constraints, **i** INTENSIVE, **j** CHROM, **k** CICA with periodicity constraint, **l** CICA with periodicity and chrominance constraints

To assess the effect of the combination of periodicity and chrominance constraints on the cICA algorithm, and its utility in fitness scenarios, three videos were recorded on a fitness bike. They are categorized as LIGHT, MODERATE and INTENSIVE based on the speed of motion and intensity of training. Figure 4 shows a snapshot from the three videos which exhibit a prominent periodic motion along with accuracy comparisons between recovered rPPG from CHROM, cICA using only the periodicity constraint and cICA using both the periodicity and the chrominance constraint. The CHROM method was able to extract the correct rPPG signal for the LIGHT video (Fig. 4b) whereas cICA with only the periodicity constraint converged to the component representing the strong periodic motion (Fig. 4c). As expected, the combination of the CHROM and periodicity constraints resulted in convergence to the correct rPPG signal (Fig. 4d).

However, the CHROM method was not able to extract the correct component for the MEDIUM and INTENSIVE videos where the variations due to motion overwhelm the rPPG variations (Fig. 4f). Interestingly, where both CHROM and cICA with just the periodicity constraint failed separately, their combination resulted in a partial convergence to the rPPG signal as is visible in Fig. 4h. This can be attributed to the constriction of the solution space resulting in the optimizer to converge to the correct rPPG signal.

Finally, for the INTENSIVE video, the motion component was much stronger and none of the methods succeeded in extracting the rPPG signal (Fig. 4j–l). This calls for the use of a motion compensation scheme to mitigate such high intensity motions.

Consequently, these two constraints, the autocorrelation being a bit too lenient and the chrominance based constraint being too restrictive, in choosing the best weighting matrix, can be combined to guide the optimizer in choosing a weighting matrix with optimum flexibility. The combination is also advantageous for fitness scenarios with limited periodic motion, without any motion compensation. This implementation showed improved results compared to both ICA and CHROM methods the analysis of which is presented in “Results and discussion” section. The use of these two constraints in our framework is described in the next subsection.

### Constrained ICA

A generic contrast function for ICA as defined by [8], is the negentropy function given by $J(\mathbf {y})=H(\mathbf {y}_{gauss})-H(\mathbf {y})$ where *H*(.) is the differential entropy and $\mathbf {y}_{gauss}$ is a random variable with a variance equal to that of the output signal $\mathbf {y}$. In FastICA [8], an approximation of the negentropy was introduced for more reliability and flexibility given by

8 $$\begin{aligned} J(\mathbf {y})\thickapprox \rho \left[ E\{G(\mathbf {y})\}-E\{G(v)\}\right] ^{2} \end{aligned}$$

where $\rho$ is a positive constant, *v* is a zero mean, unit variance Gaussian and G(.) can be any non-quadratic function. As suggested by [28] a good general purpose function is given by

9 $$\begin{aligned} G(\mathbf {y})=\frac{\log \cos (a\mathbf {y})}{a} \end{aligned}$$

with $1<a<2$. Constrained ICA aims to alleviate the issues of ICA with the help of Lagrange multiplier methods. Lagrange multiplier methods [29] are a tool for performing constrained optimization problems following the general form

10 $$\begin{aligned} \mathrm{minimize}\,f\mathbf{(X)},\,\mathrm{subject\,to}\,g\mathbf{(X)}\le 0,\quad h\mathbf{(X)}=0 \end{aligned}$$

where $f(\mathbf {X})$ is the objective function, $g(\mathbf {X})$ is a set of inequality constraints and $h(\mathbf {X})$ is a set of equality constraints.

The objective of obtaining the weighting matrix to give the optimum cardiac pulse using cICA can be fulfilled with the help of the inequality constraint

11 $$\begin{aligned} g(\mathbf {w})=\epsilon (\mathbf {w})-\zeta \le 0 \end{aligned}$$

where **w** represents a single demixing weight vector of size equal to the number of input channels and $\epsilon (\mathbf {w})$ represents the set of constraints to be satisfied. The optimum $\mathbf {w}$ then extracts the optimum cardiac pulse using $\mathbf {y}=\mathbf {w}^{\mathrm {T}}\mathbf {x}$. Using average of squared autocorrelation as a constraint gives $g(\mathbf {w})$ as

12 $$\begin{aligned} g_1(\mathbf {w})=\zeta _1-E\{\mathbf {r}^{2}\}\le 0 \end{aligned}$$

where $\zeta _1$ denotes the threshold for the lower bound of the optimum autocorrelation. This constraint guides the optimizer towards choosing the weighting matrix that results in a signal of high periodicity, with the minimum expectation of its mean squared autocorrelation as $\zeta _1$. Next, the CHROM constraint is defined as

13 $$\begin{aligned} g_2(\mathbf {w})=\left\| \mathbf {w}-\mathbf {w_{chrom}}\right\| -\zeta _2\le 0 \end{aligned}$$

where $\mathbf {w_{chrom}}$ is the CHROM weighting matrix from Eq. 7, and $\zeta _2$ is the threshold for the upper bound for discrepancy between the optimum and the CHROM weighting vectors. This constraint guides the optimizer to converge towards the CHROM weighting matrix $\mathbf {w_{chrom}}$.

### cICA optimization algorithm

The general cICA problem can be defined as [9]

14 $$\begin{aligned} \mathrm {Maximize}: &\quad J(\mathbf {y}) = \rho \left[E\{G(\mathbf {w^{\mathrm {T}}x})\}-E\{G(v)\}\right]^{2},\nonumber \\ \mathrm {Subject\,to}: &\quad g_i(\mathbf {w}) \le 0,\,h(\mathbf {w})=E\{\mathbf {y^{2}}\}-1=0 \end{aligned}$$

where $J(\mathbf {y})$ is the one-unit contrast function as defined in Eq. 8, $g_i(\mathbf {w})$ is the set of inequality constraints to be satisfied from Eqs. 12 and 13, and $h(\mathbf {w})$ constrains the output $\mathbf {y}$ to have unit variance.

The augmented Lagrangian formulation as adapted from [9] was used primarily because of its robustness owing to the use of penalty parameters to maintain the convexity assumption [29].

15 $$\begin{aligned} \mathcal {L}(\mathbf {w},\mu ,\lambda )= & {} J(\mathbf {y})-\frac{1}{2\gamma _i}\left[ \left\{ \left[max\{0,\bar{g_i}(\mathbf {w})\}\right]^{2}-\mu _{i}^{2}\right\} \right] \nonumber \\ \\&\quad \quad-\lambda h(\mathbf {w})+\frac{1}{2}\beta \left\| h(\mathbf {w})\right\| ^{2} \end{aligned}$$

where $\bar{g_i}(\mathbf {w})=\mu _i+\gamma _i g_i(\mathbf {w})$, $\mu _i$ and $\lambda$ are the Lagrange multipliers corresponding to $g_i(\mathbf {w})$ and $h(\mathbf {w})$ respectively. $\left\| \cdot \right\|$ denotes the Euclidean norm and the terms $\frac{1}{2}\gamma _i\left\| \cdot \right\| ^{2}$ and $\frac{1}{2}\beta \left\| \cdot \right\| ^{2}$ are the penalty terms that makes sure that the optimization problem is held at the condition of local convexity assumption: $\nabla _{xx}^{2}\mathcal {L>}0$, $\gamma _i$ and $\beta$ being the constraint-wise penalty parameters.

The first derivative of $\mathcal {L}$ w.r.t $\mathbf {w}$ required for the optimization given by

16 $$\begin{aligned} \mathcal {L}_{w}^{'}=\bar{\rho }E\{\mathbf {x}G_{y}^{'}(\mathbf {y})\}-\frac{1}{2}\mu E\{g_i^{'}(\mathbf {w})\}-\lambda E\{\mathbf {xy}\} \end{aligned}$$

where $\bar{\rho }=\pm \rho$ depending on the sign of $E\{G(\mathbf {y})\}-E\{G(v)\}$, $G_{y}'(\mathbf {y})$ and $g_i'(\mathbf {w})$ are the first derivatives of $G(\mathbf {y})$ and $g_i(\mathbf {w})$ w.r.t $\mathbf {y}$ and $\mathbf {w}$ respectively. The Hessian $\mathcal {L}_{\mathbf {w}_{k}}^{''}$ is calculated as

17 $$\begin{aligned} \mathcal {L}_{\mathbf {w}_{k}}^{''}=\bar{\rho }\mathbf {R}_{\mathbf {xx}}E\{G_{y}^{''}(\mathbf {y})\}-\frac{1}{2}\mu E\{g_{i}^{''}(\mathbf {w})\}-\lambda \end{aligned}$$

the inversion of which is not problematic because $\mathbf {R_{xx}}$ being the covariance matrix of the whitened and centered signal $\mathbf {x}$ is an identity matrix. $G_{y^{2}}^{''}(\mathbf {y})$ and $g_{i}^{''}(\mathbf {w})$ are second order derivatives and $\mathcal {L}_{\mathbf {w}_{k}}^{''}$ is of size $m\times m.$ The first and second derivatives of autocorrelation in Eq. 12 are not trivial and are presented in the appendix to maintain structure.

The expectation in the equations were calculated by using all the samples of the input signal $\mathbf {x}.$ The first and second derivatives were then fed into the fmincon function of MATLAB using the interior point algorithm [30] to obtain the final weighting matrix $\mathbf {w}^*$ which was then used to obtain the final rPPG signal.

### System framework

**Fig. 5:**
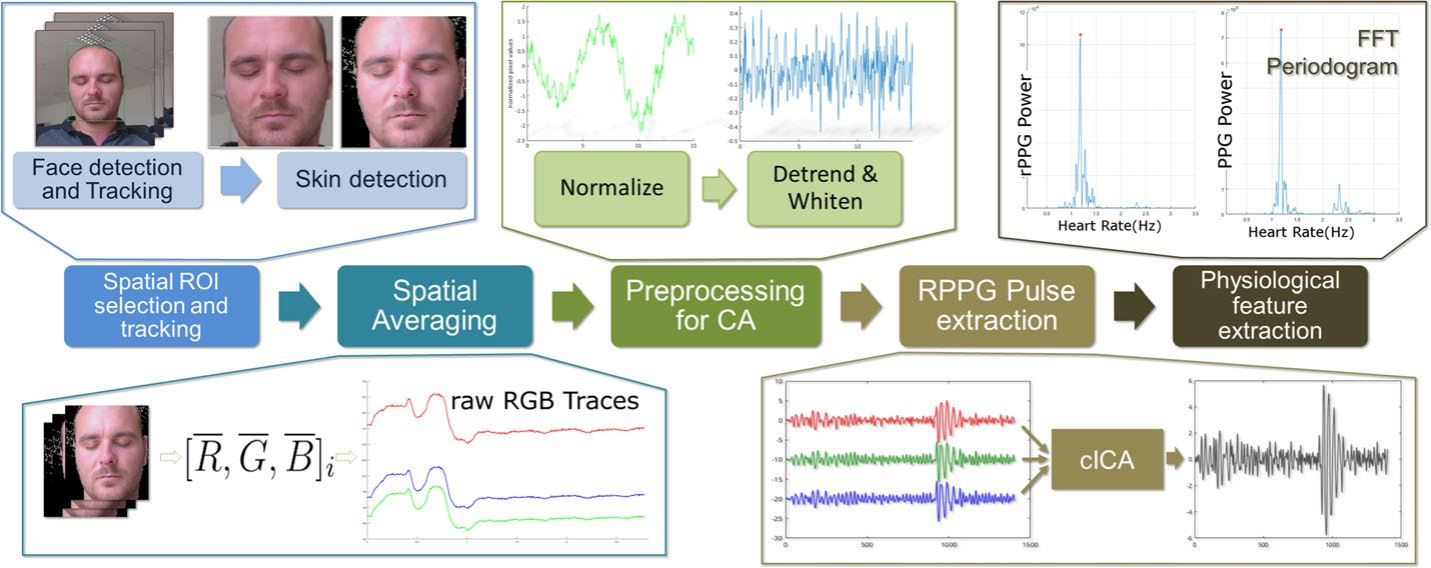
Flowchart of the proposed method

Figure 5 depicts the entire workflow of the procedure. Let $\mathbf {x}=[x_{1}\,x_{2}\,x_{3}]^{T}$ be the temporal RGB traces with each $x_{m},\,m\in [1\cdots3]$ corresponding to the channel-wise temporal trace vector of size *N*. Each $x_m,$corresponding to each video frame, was obtained by spatial averaging of the skin pixels from the face. These candidate skin pixels were obtained by using the method proposed by Conaire et al. [31] after performing face detection and tracking using the Viola–Jones and Kanade–Lucas–Tomasi implementations provided by the computer vision toolbox of MATLAB. Corner detection in the detected face was also performed for tracking to crop the face based on facial landmarks.

Next, the temporal RGB traces $\mathbf {x}$ were detrended using a smoothness priors approach by Karjalainen et al. [32] to remove any low frequency trends in the signal. Some preprocessing, generally recommended for ICA, was also performed. *Centering* was first performed to transform the obtained signal $\mathbf {y}=\mathbf {w}^T\mathbf {x}$ to a zero-mean signal, followed by *whitening* to ensure that the components were uncorrelated and their variances equaled unity. Note that whitening, which changes the standard deviation of a signal, was not performed for the CHROM constraint because the CHROM method relies on using the standard deviation of a linear combination of the RGB traces as seen in Eq. 6. These traces were then fed to the rPPG extraction module where the cICA algorithm was used to extract the window-wise rPPG signal.

Once the rPPG signal was estimated, the window-wise heart rate was calculated from the highest peak of the FFT filtered within the limits of normal heart rate $\mathcal {F} \in [0.7, 3]$ Hz over a temporal moving window using a step size of 0.5 s. ICA is generally known to work better with longer temporal signals, however, all the processing was performed over a 30 s window using the weighting matrix $\mathbf {w}_{k}$ obtained at window *k* as an initial estimate for calculation of $\mathbf {w}_{k+1}$ at the next window. This was done in order to mimic the constraints of a live scenario thereby making the analysis more relevant. The 30 s length for the window was selected as a trade-off between speed and availability for enough data for convergence. That being said, a 15 s window had to be chosen for the MMSE-HR database because of the variable and oftentimes short length of the videos. Finally, Kalman filter was applied, as it were a live scenario, to compensate for spurious outliers in the estimated heart rates. These outliers, although useful in scenarios such as emotion elicitation to extract fleeting responses, are not relevant in our study where our focus is on heart rate signal extraction. The choice of Kalman filter for smoothing is apt and useful for any rPPG extraction algorithm since there might always be spurious measurements due to motion and illumination artifacts. And since all the methods are treated with the same Kalman filter routine, the comparison between the metrics stands valid.

## Results and discussion

The cICA algorithm was validated using our in-house UBFC-RPPG database comprising of two datasets comprising of 9 (about 21k frames) and 46 (about 94k frames) videos respectively and the public MMSE-HR [1] database comprising of 97 (about 105k frames) videos. The two in-house datasets are labeled as SIMPLE and REALISTIC, respectively, and are ethically approved by the human participants. The SIMPLE dataset consists of the subjects relaxed with their eyes close with a realistic background and moderately varying light conditions. For the REALISTIC dataset, the subjects were required to play a time sensitive mathematical game that aimed at augmenting their heart rate while simultaneously emulating a normal human-computer interaction scenario.

**Fig. 6:**
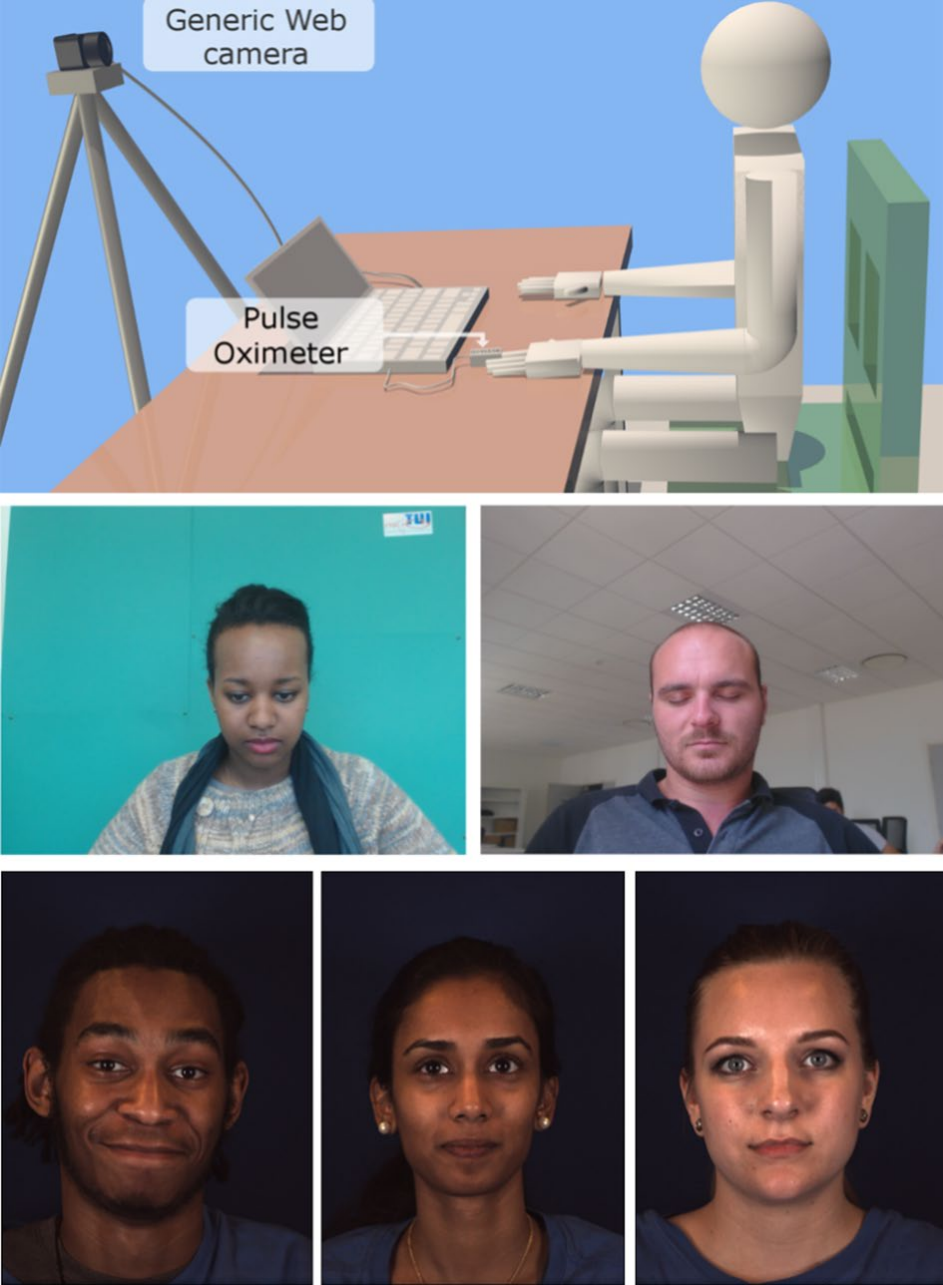
Experimental setup (top) and sample images from the UBFC-RPPG REALISTIC and SIMPLE (middle) and MMSE-HR (bottom) databases

The UBFC-RPPG database which is focused specifically on rPPG analysis was created using a custom C++ application for video acquisition with a Logitech C920 web camera placed at a distance of about 1 m from the subject. The video was recorded with a frame resolution of 640 × 480 in 8-bit uncompressed RGB format at 30 frames per second. A CMS50E transmissive pulse oximeter was used to obtain the ground truth PPG data comprising of the PPG waveform as well as the PPG heart rates. The experimental setup with sample images from both the databases is depicted in Fig. 6.

The MMSE-HR database is specifically geared towards research on emotion elicitation, but the videos are usable for rPPG analysis. Due to its focus towards emotion elicitation, the videos in this database comprise of a large number of facial expressions and movements which aids in assessing our method rigorously. Based on its working principle, rPPG signals should be ideally compared with PPG ground truth signals. However, the MMSE-HR dataset only provides ground truth heart rates, acquired using the BIOPAC 150 data acquisition system which were calculated using contact based ECG electrodes. Moreover, it was also observed that the PPG heart rates were unreliable and spurious at many instances, most definitely because of movements of the subject. These two facts do make the MAE metrics in our analysis less comprehensive. This also highlights the issue of contact based measurements, where even the ground truth signals are not entirely reliable. On the other hand, our UBFC-RPPG database provides both the PPG waveforms as well as the PPG heart rates, thus furnishing the means for a more comprehensive analysis.

Table 1 shows the performance comparisons between ICA and cICA and other state of the art methods, viz., PCA [33], Green [2], CHROM [4], POS [15], and G-R [34]. In literature, there are several works which uses ICA for rPPG extraction. However, the core algorithm for it remains the same. The ICA implementation used for our analysis has been adapted from FastICA [8]. Furthermore, the analysis of all the methods was performed using exactly the same pre and post processing steps like normalization, filtering and smoothing. The exact metrics of ICA from related state of the art methods such as [6, 7] and [5] could not be used because they all use their own private databases which were inaccessible to the public. However, as mentioned earlier, the core algorithm of ICA remains the same making the metrics in Table 1 applicable. Furthermore, comparison with smart ROI selection methods such as [17, 19, 20] and [13] was not deemed relevant in order to limit the comparison amongst source separation methods, and the fact that our method can easily be incorporated into an ROI selection framework. The best metrics per method are italicized to compare their effectiveness clearly.

**Table 1:** Performance metrics

	UBFC-RPPG						MMSE-HR	
	SIMPLE			REALISTIC				
	MAE	SNR	r	MAE	SNR	r	MAE	r
cICA	*0.62*	2.01	*0.99*	*3.14*	*− 0.75*	*0.91*	*4.69*	0.79
ICA	0.67	2.70	0.98	6.02	− 1.11	0.79	5.84	0.67
PCA	2.04	− 1.43	0.97	9.65	− 3.45	0.67	9.15	0.49
Green	9.86	− 1.61	0.29	7.73	− 2.78	0.68	10.65	0.47
CHROM	0.72	*3.04*	0.99	3.81	− 0.93	0.87	5.59	*0.83*
POS	0.67	2.57	0.99	4.73	− 1.60	0.80	5.77	0.82
G-R	0.67	1.97	0.99	9.79	− 3.10	0.65	8.56	0.58

The metrics used in our analysis are mean absolute error (MAE) in beats per minute (bpm), signal-to-noise ratio (SNR) and Pearson’s correlation coefficient (*r*) between heart rate calculated using the rPPG signal, $HR_{rPPG}$ and the heart rate calculated using the ground truth PPG waveform, $HR_{PPG}$. The MAE was calculated as the window-wise mean of $\vert HR_{rPPG}-HR_{PPG} \vert$, averaged per video. The windowed method is computationally more taxing, owing to the smaller window length, but is more realistic. The SNR (dB) was calculated as the ratio of the power of the main pulsatile component of the PPG to that of the background noise to accommodate the wide dynamic range of the signals.

However, it is to be noted that the MMSE-HR database does not provide the ground truth waveforms, thereby obliging the use of the main pulsatile component of the RPPG instead of the PPG for the SNR calculation. In this case, the SNR just represented the strength of the main pulsatile component, which in itself is a useful metric, but is not valid for the comparison with the SNR for other databases which were calculated in a different manner. As a result, the SNR values for the MMSE-HR database are not really relevant and are omitted. The MAE values, however, are relevant since it is calculated as the difference between rPPG and PPG heart rates, which are provided as ground truth for the MMSE-HR database.

It was also worth assessing the resilience of the cICA algorithm against changes in parameters such as image resolution and window length. This analysis was performed in two parts. The MMSE-HR database was chosen to assess the effect of changes in image resolution since it provides images of resolution 1040 × 1392 which offer the possibility to asses scaled down versions of the frames, to 75, 35% and even to 10%, in favor of the UBFC-RPPG database which provides images of a lower resolution, viz. 640 × 480 pixels. On the other hand, due to the shorter duration of videos in the MMSE-HR database, the UBFC-RPPG REALISTIC dataset was chosen to assess the effect of changes in the window length, the assessment being done against window lengths of 10 and 20 s, respectively.

Table 2 lists the average results of this assessment over the three datasets. The metrics in the last row correspond to the original results. It was observed that there was a slight decline in the performance with respect to the window length of 10 s, which is expected, owing to the lack of enough data for the ICA objective function to establish independence. Correspondingly, frames of lower resolution reflect loss of spatial information. Even though this loss is in itself not too deteriorating for the signal quality, the fact that it was coupled with the 15 s window length for the MMSE-HR database, explains the slightly higher MAE values for frames scaled down to 35 and 70%. As expected, the amount of information loss is slightly more pronounced at a scale of 10%, depicting the slight decline in the quality of the extracted rPPG signal with reduction in resolution. The decline due to loss of spatial information can be attributed to the reduction in the total number of skin pixels used to get the average signal value per frame.

**Table 2:** Effect of window length and scale

Window length (s)	UBFC-RPPG REALISTIC		
	MAE	SNR	r
10	6.19	− 1.34	0.72
20	4.09	− 1.18	0.83
30	3.14	− 0.78	0.91

Scale (%)	MMSE-HR	
	MAE	r
10	10.84	0.38
35	7.06	0.57
75	6.68	0.61
100	4.69	0.79

**Fig. 7:**
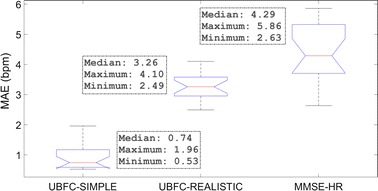
Box plot of MAE from cICA over 20 observations for the two databases

Finally, since cICA is essentially an optimization algorithm where the weights are randomly initialized, it was worth assessing its consistency over multiple runs. Figure 7 shows the box plot comparing the MAEs with cICA for the UBFC-SIMPLE dataset without much movement under ambient light, the UBFC-REALISTIC dataset with subjects working on a computer under ambient light, and the MMSE-HR dataset with subjects exhibiting facial expressions under indoor lighting. These tests on the datasets were performed 20 times. It is visible from the box plot that the cICA method performs consistently resulting in MAEs in the range [0.53, 1.96] bpm, [2.49, 4.1] bpm and [2.63, 5.86] bpm for the UBFC-SIMPLE, UBFC-REALISTIC and MMSE-HR datasets respectively.

**Fig. 8:**
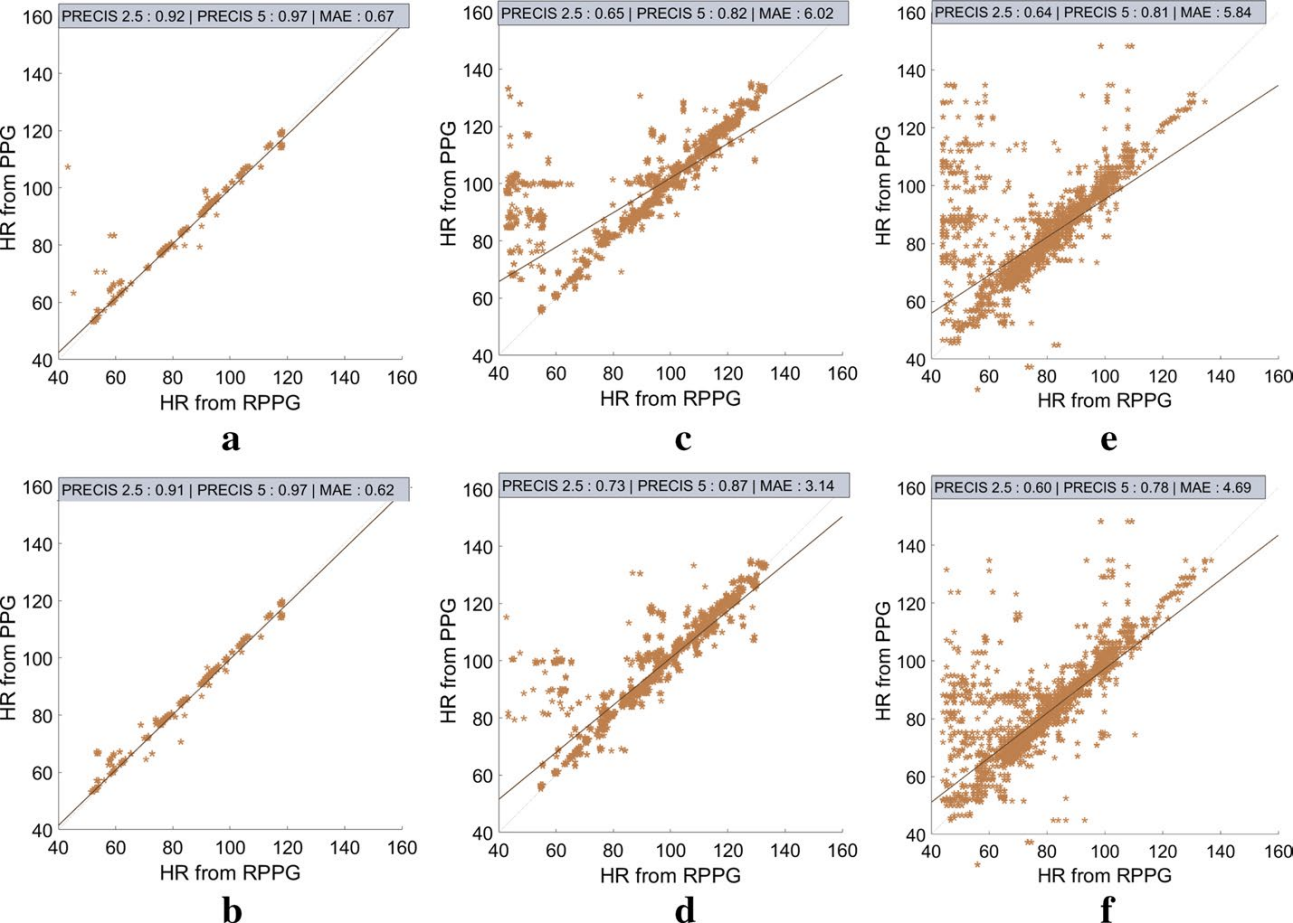
Correlation comparison plots for ICA vs cICA. **a** ICA for UBFC-RPPG/SIMPLE, **b** cICA for UBFC-RPPG/SIMPLE, **c** ICA for UBFC-RPPG/REALISTIC, **d** cICA for UBFC-RPPG/REALISTIC, **e** ICA for MMSE-HR, **f** cICA for MMSE-HR

The global correlation analysis using window-wise calculations between HRs from PPG versus RPPG obtained from all the videos in each dataset for the skin-segmented pixel data for one particular run is presented in Fig. 8. The metrics PRECIS 2.5 and PRECIS 5 show the percentage of windows where $\delta =\vert HR_{rPPG}-HR_{PPG} \vert <2.5$ and 5 bpm respectively. *n* represents the total number of windows used in the analysis, *r* is Pearson’s correlation coefficient and *y* depicts the equation of the fitted line. It is worth mentioning that the MAE values in Fig. 8 and Table 1 differ from those in Fig. 7, which are averaged over 20 executions, but are obviously in range. Moreover, differences in range of $10^{-2}$ bpm are inconsequential.

The analysis of the SIMPLE dataset, as the name suggests, was quite easy for both ICA and cICA. However, cICA did remove the few outliers that are present in the case of ICA. This is reflected in the low MAE and high SNR values as shown in Table 1. This can be attributed to the fact that the subjects were generally relaxing, mostly with their eyes closed, which resulted in minimal motion artifacts. On the other hand, the REALISTIC dataset was slightly more challenging since the subjects were actually working on the computer and were only requested to keep their hand still for the PPG sensor. Similarly, the MMSE-HR database was challenging owing to it being an emotion elicitation database. There were many instances where the subjects laughed out loud, exhibited considerable movements, while regularly manifesting various facial expressions. This resulted in the usual problems arising from movement of the subjects and the face ROIs. Consequently, the presence of outliers was more pronounced for both the REALISTIC dataset and the MMSE-HR database, which cICA was able to reduce. Also, the fitting line was closer to the 45° line with cICA as compared to ICA.

## Conclusions and future work

In this paper we presented a novel semi blind source separation method for the application of rPPG measurements using autocorrelation and chrominance based constraints to guide the ICA separation process. The cICA using autocorrelation and chrominance constraints provides better result than simple ICA while removing the extra step for choosing the best component. The periodogram of the extracted signals was also consistently closer to that of the PPG.

The inclusion of the chrominance constraint can also aid for rPPG measurement in scenarios comprising of periodic movements. Since in this case, the autocorrelation constraint is likely to be contaminated by the signal corresponding to the periodic movement, the optimizer can favor the signal satisfying both, the autocorrelation and the CHROM constraints. Furthermore, for improving accuracy, better face and skin detectors and trackers can be investigated. Also, even though the CHROM constraint helps the convergence to the correct rPPG signal for limited movements, it does fail when they are more pronounced in speed and intensity. The method can thus benefit with motion compensation which itself is another interesting subject for research. Last, but not the least, we average the entire skin segmented image to obtain a single value and thus loose any spatial information. Higher order analysis which preserves the spatial relationships between pixel neighborhoods is also an important avenue to look into.

## Appendix

### Derivatives of the autocorrelation constraint

Here we present the first and second derivatives of the autocorrelation constraint, $g_{1}(w)$ needed by the optimization algorithm. We follow the convention that the derivative of a scalar w.r.t a column vector is a column vector of the same size as that of the vector. The first derivative of $g_{1}(w)$ in Eq. 12 can be obtained as follows. Considering squared autocorrelation as $\mathbf {r}^{2}=\left[r_{1}^{2}\,r_{2}^{2} \cdots r_{N}^{2}\right]$,

18 $$\begin{aligned} g_{1}'(\mathbf {w})&=-\,E\left\{\frac{\partial }{\partial \mathbf {w}}\left( \begin{bmatrix}r_{1}^{2}&r_{2}^{2} &\cdots& r_{N}^{2} \end{bmatrix}\right) \right\} \end{aligned}$$

where the derivative of the squared autocorrelation $\mathbf {r}^{2}$ is then obtained using the chain rule of derivatives. Also, we know that $\mathbf {y}=\mathbf {w}^{T}\mathbf {x}$ giving $\frac{\partial \mathbf {y}}{\partial \mathbf {w}}=\mathbf {x}$. Expanding on the derivative of $\mathbf {r}^{2}$, we have

19 $$\begin{aligned} \frac{\partial (\mathbf {r}^{2})}{\partial \mathbf {w}}= {} \mathbf {x}\frac{\partial (\mathbf {r}^{2})}{\partial \mathbf {y}}\end{aligned}$$

20 $$\begin{aligned}= {} \mathbf {x} \begin{bmatrix} \frac{\partial (r_{1}^{2})}{\partial y_{1}}\quad\frac{\partial (r_{2}^{2})}{\partial y_{1}}\quad\cdots\quad\frac{\partial (r_{N}^{2})}{\partial y_{1}}\\ \frac{\partial (r_{1}^{2})}{\partial y_{2}}\quad\frac{\partial (r_{2}^{2})}{\partial y_{2}}\quad\cdots\quad\frac{\partial (r_{N}^{2})}{\partial y_{2}}\\ \vdots\quad\vdots\quad\ddots\quad\vdots \\ \frac{\partial (r_{1}^{2})}{\partial y_{N}}\quad\frac{\partial (r_{2}^{2})}{\partial y_{N}}\quad\cdots\quad\frac{\partial (r_{N}^{2})}{\partial y_{N}} \end{bmatrix}\end{aligned}$$

21 $$\begin{aligned}= {} \mathbf {x} \begin{bmatrix} 2r_{1}\frac{\partial r_{1}}{\partial y_{1}}\quad\cdots\quad2r_{N}\frac{\partial r_{N}}{\partial y_{1}}\\ \vdots\quad\ddots\quad\vdots \\ 2r_{1}\frac{\partial r_{1}}{\partial y_{N}}\quad\cdots\quad2r_{N}\frac{\partial r_{N}}{\partial y_{N}} \end{bmatrix}\end{aligned}$$

22 $$\begin{aligned}= {} 2\mathbf {x}\begin{bmatrix}r_{1}\frac{\partial r_{1}}{\partial y_{1}}\quad\cdots\quad r_{N}\frac{\partial r_{N}}{\partial y_{1}}\\ \vdots\quad\ddots\quad \vdots \\ r_{1}\frac{\partial r_{1}}{\partial y_{N}}\quad\cdots\quad r_{N}\frac{\partial r_{N}}{\partial y_{N}} \end{bmatrix} \end{aligned}$$

The size of $\frac{\partial (\mathbf {r}^{2})}{\partial \mathbf {w}}$ is then $3\times N$ from the product of $\mathbf {x}_{3\times N}$ with the Jacobian of size $N\times N$. Consequently, its expectation ends up having a size of $3\times 1$ since it is nothing but a temporal mean over *N* samples. The Jacobian in Eq. 23 can be concisely expressed as $$\begin{bmatrix}r_{1}\frac{\partial r_{1}}{\partial \mathbf {y}}&r_{2}\frac{\partial r_{2}}{\partial \mathbf {y}}&\cdots&r_{N}\frac{\partial r_{N}}{\partial \mathbf {y}}\end{bmatrix}$$ where each column is the product of the derivative $\frac{\partial r_{1}}{\partial \mathbf {y}}$ and the scalar $r_{k}$ and is of size $N\times 1.$ Deriving Eq. 4, $r_{k}=\mathbf {y}T_{k}\mathbf {y}^{T}$, listed here for convenience, w.r.t $\mathbf {y}$ using the product rule of differentiation, 2

23 $$\begin{aligned} \frac{\partial r_{k}}{\partial \mathbf {y}}= {} \mathbf {y}\frac{\partial }{\partial \mathbf {y}}\left(T_{k}\mathbf {y}^{T}\right)+\mathbf {y}T_{k}\frac{\partial }{\partial \mathbf {y}}\left(\mathbf {y}^{T}\right)\nonumber \\= {} \mathbf {y}\frac{\partial }{\partial \mathbf {y}}\left(\mathbf {y}T_{k}^{T}\right)+\mathbf {y}T_{k}\nonumber \\= {} \mathbf {y}T_{k}^{T}+\mathbf {y}T_{k}=\mathbf {y}\left(T_{k}^{T}+T_{k}\right) \end{aligned}$$

where $\frac{\partial }{\partial \mathbf {y}}(T_{k}\mathbf {y}^{T})=T_{k}^{T}$ comes from the fact that the differential of $T_{k}\mathbf {y}^{T},$ a vector, will remain the same even when it is transposed and the derivative is computed element-wise. For conciseness, we will represent the sum $T_{k}+T_{k}^{T}$ as $\mathbf {T}_{k}$. Finally to be consistent with our convention, using the same argument of the differential being immutable to transpositions, the row vector $\frac{\partial r_{k}}{\partial \mathbf {y}}$ can be transposed into a column vector and the matrix $\frac{\partial \mathbf {r}}{\partial \mathbf {y}}$ can be built as

24 $$\begin{aligned} \frac{\partial \mathbf {r}}{\partial \mathbf {y}}=\begin{bmatrix}r_{1}\mathbf {T}_{1}\mathbf {y}^{T}&\cdots&r_{N}\mathbf {T}_{N}\mathbf {y}^{T}\end{bmatrix} \end{aligned}$$

giving $g_{1}'(\mathbf {w)}$ in Eq. 19 as

$$\begin{aligned} g_{1}'(\mathbf {w})=-\,2\mathbf {x}E\left\{ \begin{bmatrix} r_{1}\mathbf {T}_{1}\mathbf {y}^{T}&\cdots&r_{N}\mathbf {T}_{N}\mathbf {y}^{T} \end{bmatrix}\right\} \end{aligned}$$

which can be further simplified to

25 $$\begin{aligned} g_{1}'(\mathbf {w})=-\,2\mathbf {x}\left[ \begin{array}{ccc} \mathbf {T}_{1}\mathbf {y}^{T}&\cdots&\mathbf {T}_{N}\mathbf {y}^{T} \end{array}\right] \mathbf {r}^{T}/N \end{aligned}$$

Since the expectation is a temporal mean the element-wise multiplication with $r_{k}$ can be replaced by multiplication with the vector $\mathbf {r}^{T}$ which also simplifies the computation.

Next, to simplify the calculation of the second derivative of $g(\mathbf {w})$ , we perform column-wise matrix multiplication in Eq. 26, omitting the scalar multiplication and division, to obtain

26 $$\begin{aligned} g_{1}'(\mathbf {w})= & {} -\mathbf {x}\left[ \begin{array}{ccc} \mathbf {T}_{1}r_{1}\mathbf {y}^{T}+\cdots+\mathbf {T}_{N}r_{N}\mathbf {y}^{T} \end{array}\right] \end{aligned}$$

27 $$\begin{aligned}= & {} -\mathbf {x}\sum _{\begin{array}{c} {k=1} \end{array} }^{{N}}\mathbf {T}_{k}r_{k}\mathbf {y}^{T} \end{aligned}$$

And since differentiation and summation are interchangeable based on the sum rule, $g''(w)$ can be obtained by

28 $$\begin{aligned} g_{1}''(\mathbf {w})= & {} -\mathbf {x}\sum _{\begin{array}{c} {k=1} \end{array} }^{{N}}\frac{\partial (\mathbf {T}_{k}r_{k}\mathbf {y}^{T})}{\partial \mathbf {w}}\end{aligned}$$

29 $$\begin{aligned}= {} -\mathbf {x}\sum _{\begin{array}{c} {k=1} \end{array} }^{{N}}\frac{\partial \left(\mathbf {T}_{k}r_{k}\mathbf {y}^{T}\right)}{\partial \mathbf {y}}\frac{\partial \mathbf {y}}{\partial \mathbf {w}}\end{aligned}$$

30 $$\begin{aligned}= {} -\mathbf {x\left( \sum _{\begin{array}{c} {k=1} \end{array} }^{{N}}\frac{\partial \left({\mathbf {T}_{k}} r_{k} \mathbf {y}^{T}\right)}{\partial \mathbf {y}}\right) }\mathbf {x}^{T} \end{aligned}$$

The derivative of $\mathbf {T}_{k}r_{k}\mathbf {y}^{T}$ w.r.t $\mathbf {y}$ is then obtained by the product rule of differentiation.

31 $$\begin{aligned} \frac{\partial \left(\mathbf {T}_{k}r_{k}\mathbf {y}^{T}\right)}{\partial \mathbf {y}}= {} \mathbf {T}_{k}\frac{\partial r_{k}}{\partial \mathbf {y}}\mathbf {y}^{T}+\mathbf {T}_{k}r_{k}\end{aligned}$$

32 $$\begin{aligned}= {} \mathbf {T}_{k}\left( \frac{\partial r_{k}}{\partial \mathbf {y}}\mathbf {y}^{T}+r_{k}\right) \end{aligned}$$

which is of size $N\times N$. Consequently, the size of $g_{1}''(\mathbf {w})$ turns out to be $3\times 3$ since the sum of $\frac{\partial (\mathbf {T}_{k}r_{k}\mathbf {y}^{T})}{\partial \mathbf {y}}$ over *N* samples is also of size $N\times N$. $g_{1}''(\mathbf {w})$ is further used in the Lagrange multipliers method for cICA , implemented as an augmented lagrangian method, presented in “Constrained ICA” section.

## Footnotes

1 https://sites.google.com/view/ybenezeth/ubfcrppg.
